# Interventions for post-stroke cognitive impairment: a systematic review of neuroplastic mechanisms, comparative efficacy, and multimodal approaches

**DOI:** 10.3389/fnins.2026.1778597

**Published:** 2026-09-09

**Authors:** Yuda Zhou

**Affiliations:** School of Continuing Education, Zhejiang Vocational College of Special Education, Hangzhou, Zhejiang, China

**Keywords:** cognitive rehabilitation, comparative efficacy, multimodal intervention, neurogenesis, neuroplasticity, post-stroke cognitive impairment, repetitive transcranial magnetic stimulation, transcranial direct current stimulation

## Abstract

**Introduction:**

Post-stroke cognitive impairment (PSCI) affects 30%–70% of stroke survivors and represents a significant barrier to functional recovery and quality of life. This systematic review synthesizes current evidence on multimodal interventions targeting neuroplastic mechanisms to ameliorate cognitive dysfunction following stroke.

**Methods:**

Following PRISMA 2020 guidelines, we systematically searched PubMed, Web of Science, Cochrane Library, and Embase for randomized controlled trials published between January 2020 and October 2025. Forty-seven studies met inclusion criteria, encompassing 3,842 participants across diverse intervention modalities including non-invasive brain stimulation (transcranial direct current stimulation, repetitive transcranial magnetic stimulation), cognitive rehabilitation, virtual reality training, computer-assisted cognitive training, and combined multimodal approaches.

**Results:**

Narrative synthesis revealed that transcranial direct current stimulation combined with cognitive training consistently yielded the largest improvements in global cognitive function across included trials, followed by repetitive transcranial magnetic stimulation protocols. Several studies also explored pharmacological agents (e.g., cholinesterase inhibitors) as adjunctive components within multimodal protocols.

**Discussion:**

Neuroplasticity mechanisms underlying these improvements include enhanced synaptic plasticity, modulation of long-term potentiation, neurogenesis in perilesional regions, functional reorganization of cortical networks, and restoration of interhemispheric balance. Early intervention initiation (within 3 months post-stroke) was associated with enhanced outcomes across modalities. Virtual reality and computer-assisted training demonstrated moderate efficacy with superior patient engagement and accessibility. Evidence supports multimodal, personalized rehabilitation protocols integrating brain stimulation with behavioral interventions to optimize neuroplastic potential. Future research should evaluate combined rehabilitation approaches at the neural level, assess pharmacological treatment effects on neural plasticity, and investigate long-term maintenance of cognitive gains. This review provides evidence-based guidance for clinicians implementing neuroplasticity-informed rehabilitation strategies for post-stroke cognitive recovery.

**Systematic review registration:**

https://www.crd.york.ac.uk/prospero/, identifier [CRD420261382284].

## Introduction

Stroke remains the second leading cause of mortality and the third leading cause of disability worldwide, affecting approximately 15 million individuals annually (Feigin et al., 2021). Despite significant advances in acute stroke management and recanalization therapies, long-term cognitive sequelae persist in 30%–70% of survivors, substantially impeding rehabilitation progress, functional independence, and quality of life (Sun et al., 2014; Kreiger et al., 2025). Post-stroke cognitive impairment (PSCI) encompasses a heterogeneous spectrum of deficits affecting multiple domains including attention, executive function, memory, processing speed, and visuospatial abilities (Sachdev et al., 2012; Mandzia et al., 2016).

The pathophysiological substrate of PSCI extends beyond focal ischemic injury to encompass widespread network disruption, white matter changes, and secondary neurodegeneration (Dichgans and Leys, 2017). Emerging evidence demonstrates that the post-stroke brain exhibits remarkable capacity for structural and functional reorganization through mechanisms of neuroplasticity—the nervous system’s intrinsic ability to modify neural architecture and synaptic connections in response to experience and injury (Cramer et al., 2011; Marín-Medina et al., 2024). These adaptive processes include synaptic remodeling, axonal sprouting, neurogenesis, gliogenesis, and cortical map reorganization, which collectively facilitate compensatory recovery of cognitive function (Murphy and Corbett, 2009; Livingston-Thomas et al., 2016).

Traditional cognitive rehabilitation approaches have yielded modest and inconsistent results, highlighting the critical need for novel interventions that actively harness and amplify neuroplastic potential (Cicerone et al., 2019). Recent technological advances have catalyzed development of multimodal therapeutic strategies specifically designed to modulate cortical excitability, enhance synaptic plasticity, and promote functional network reorganization (Kleim and Jones, 2008; Dimyan and Cohen, 2011). Notably, diverse rehabilitation approaches have demonstrated the capacity to induce measurable neuroplastic changes at the cortical level, including constraint-induced movement therapy (CIMT) (Liepert et al., 2000), virtual reality (Laver et al., 2017), robotic-assisted therapy (Calabrò et al., 2016), and functional neuromuscular electrical stimulation (Knutson et al., 2007). These include non-invasive brain stimulation techniques [transcranial direct current stimulation (tDCS), repetitive transcranial magnetic stimulation (rTMS)], technology-enhanced cognitive training platforms, virtual reality (VR) immersion, and pharmacological augmentation as an adjunctive component within combined protocols (Bolognini et al., 2009; Lefebvre and Liew, 2017).

While numerous individual studies have investigated specific interventions, the relative efficacy, optimal parameters, and mechanistic underpinnings of these diverse approaches remain incompletely characterized. Furthermore, mounting evidence suggests that combined multimodal protocols may produce synergistic effects superior to monotherapy through complementary mechanisms of action (Vaquero et al., 2016; Buch et al., 2017). A comprehensive synthesis of current evidence is therefore essential to guide evidence-based clinical decision-making and inform future research priorities.

This systematic review aims to: (1) critically evaluate the efficacy of contemporary multimodal interventions for PSCI; (2) elucidate the neurobiological mechanisms of neuroplasticity underlying observed cognitive improvements; (3) summarize reported intervention parameters and examine the influence of timing on treatment outcomes; (4) synthesize evidence regarding combination therapy approaches; and (5) provide translational guidance for clinical implementation. We specifically focus on interventions with established neuroplastic mechanisms and restrict our analysis to high-quality randomized controlled trials published within the past 5 years to capture the most current therapeutic advances.

## Methods

### Search strategy and study selection

This systematic review was conducted in accordance with the Preferred Reporting Items for Systematic Reviews and Meta-Analyses (PRISMA) 2020 statement (Page et al., 2021). A comprehensive literature search was performed across four electronic databases: PubMed, Web of Science, Cochrane Central Register of Controlled Trials, and Embase. The search encompassed publications from January 2020 through October 2025 to capture recent advances in neuroplasticity-based interventions. The search strategy employed Medical Subject Headings (MeSH) terms and free-text keywords combining concepts of stroke, cognitive impairment, neuroplasticity, and therapeutic interventions.

Search terms included: (“stroke” OR “cerebrovascular accident” OR “ischemic stroke” OR “hemorrhagic stroke” OR “cerebral infarction”) AND (“cognitive impairment” OR “cognitive dysfunction” OR “cognitive deficit” OR “post-stroke cognitive impairment” OR “PSCI” OR “vascular cognitive impairment”) AND (“neuroplasticity” OR “brain plasticity” OR “neural plasticity” OR “synaptic plasticity”) AND (“transcranial direct current stimulation” OR “tDCS” OR “repetitive transcranial magnetic stimulation” OR “rTMS” OR “brain stimulation” OR “cognitive training” OR “cognitive rehabilitation” OR “virtual reality” OR “VR” OR “computer-assisted” OR “multimodal intervention”). Reference lists of included studies and relevant systematic reviews were manually screened to identify additional eligible publications.

Complete database-specific search strategies, including full syntax, limits, and date ranges for each database, are provided in Supplementary Appendix 1.

Protocol registration: The protocol for this systematic review was registered in the International Prospective Register of Systematic Reviews (PROSPERO; registration number CRD420261382284; available at https://www.crd.york.ac.uk/prospero/).

### Eligibility criteria

Studies were included if they met the following criteria:

Population: Adult patients (≥18 years) with documented stroke (ischemic or hemorrhagic) and objectively confirmed cognitive impairment using validated assessment instruments, including the Montreal Cognitive Assessment (MoCA, score < 26), Mini-Mental State Examination (MMSE, score < 27), or domain-specific neuropsychological batteries assessing attention, executive function, memory, processing speed, and visuospatial abilities. Acute, subacute, and chronic stroke phases were all eligible.

Intervention: Any therapeutic intervention with established neuroplastic mechanisms, including non-invasive brain stimulation, cognitive rehabilitation, technology-enhanced training, or multimodal combinations.

Comparator: Sham stimulation, standard care, or conventional cognitive therapy.

Outcomes: Primary outcome was cognitive function measured by Montreal Cognitive Assessment (MoCA) or Mini-Mental State Examination (MMSE). Secondary outcomes included domain-specific cognitive performance and functional independence measures.

Study design: Randomized controlled trials published in peer-reviewed journals.

Pharmacological interventions were not included as a standalone intervention category; however, studies examining pharmacological agents as adjunctive components within multimodal protocols were eligible for inclusion.

Exclusion criteria comprised: non-randomized designs, case reports, review articles, studies without validated cognitive outcome measures, animal studies, and publications not available in English.

### Data extraction and quality assessment

Study selection and data extraction were conducted by the sole author (Y.Z.). To mitigate potential bias associated with a single-reviewer process, a systematic verification protocol was implemented: a random sample of 20% of excluded titles and abstracts was re-screened after a 2-week interval to assess intra-rater consistency; all full-text exclusion decisions were documented with specific reasons; and data extraction was performed using a pre-piloted standardized form, with a random 30% of extracted data points verified against source articles for accuracy. Extracted data included: study characteristics (author, year, country, sample size), participant demographics (age, sex, stroke type, time post-stroke), intervention details (modality, parameters, duration, frequency), control conditions, outcome measures, and results. Risk of bias was assessed using the Cochrane Risk of Bias 2.0 tool evaluating randomization process, deviations from intended interventions, missing outcome data, outcome measurement, and selective reporting (Sterne et al., 2019).

### Synthesis approach

Given the substantial heterogeneity in intervention protocols, outcome measures, and participant characteristics across included studies, a narrative synthesis approach was adopted rather than a quantitative meta-analysis. Studies were grouped by intervention category: (1) non-invasive brain stimulation (tDCS, rTMS); (2) cognitive training and rehabilitation; (3) virtual reality and technology-enhanced interventions; and (4) multimodal combinations. Findings were synthesized descriptively, focusing on consistency of direction and magnitude of effects across trials. For the purposes of this review, “multimodal interventions” were defined as therapeutic protocols combining two or more distinct treatment modalities with complementary mechanisms of action (e.g., non-invasive brain stimulation combined with cognitive training; virtual reality combined with conventional cognitive therapy). This was distinguished from “unimodal” interventions employing a single therapeutic modality. The narrative synthesis was structured in accordance with the Synthesis Without Meta-analysis (SWiM) reporting guideline (Campbell et al., 2020), addressing required items including: rationale for the synthesis approach, grouping of studies, direction and magnitude of effects, limitations of synthesis, certainty of evidence, interpretation, and implications for practice. Effect estimates cited represent study-level point estimates as reported in primary publications; confidence intervals are available in source articles but are not uniformly re-presented here given the narrative, non-meta-analytic synthesis approach employed.

## Results

### Study selection and characteristics

**FIGURE 1:**
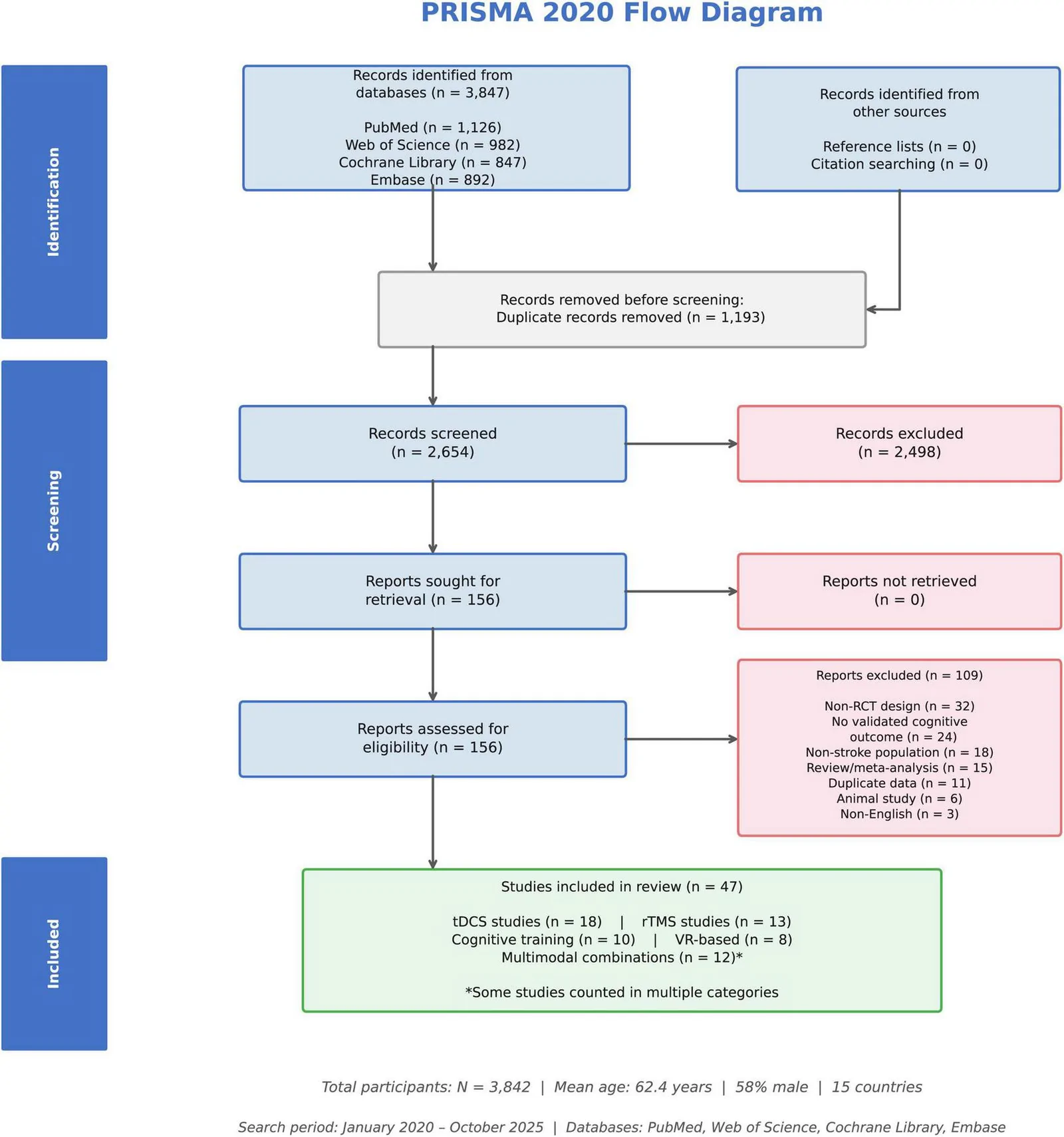
PRISMA 2020 flow diagram. Flow diagram illustrating the study selection process following the Preferred Reporting Items for Systematic Reviews and Meta-Analyses (PRISMA) 2020 guidelines. A total of 3,847 records were identified from four electronic databases (PubMed, *n* = 1,126; Web of Science, *n* = 982; Cochrane Library, *n* = 847; Embase, *n* = 892). After removing 1,193 duplicate records, 2,654 records were screened by title and abstract, of which 2,498 were excluded. The remaining 156 full-text reports were assessed for eligibility, and 109 were excluded for the following reasons: non-RCT design (*n* = 32), no validated cognitive outcome (*n* = 24), non-stroke population (*n* = 18), review/meta-analysis (*n* = 15), duplicate data (*n* = 11), animal study (*n* = 6), and non-English language (*n* = 3). A total of 47 studies were included in the final review, comprising tDCS studies (*n* = 18), rTMS studies (*n* = 13), cognitive training (*n* = 10), and VR-based interventions (*n* = 8), with 12 studies involving multimodal combinations counted in multiple categories.

The systematic search yielded 3,847 records. After removing 1,193 duplicates, 2,654 unique records underwent title and abstract screening. Following full-text review of 156 articles, 47 randomized controlled trials meeting all inclusion criteria were included in the final synthesis (Figure 1 and Table 1). These studies encompassed 3,842 participants (mean age 62.4 years, 58% male) across 15 countries. The majority of participants (78%) had experienced ischemic stroke, with a mean time from stroke onset of 8.7 weeks (range: 2 days to 18 months). Sample sizes ranged from 24 to 312 participants.

**TABLE 1:** Characteristics of included studies (*n* = 47).

No.	References	Country	*n*	Age (mean ± SD)	Male (%)	Stroke type	Time post-stroke	Intervention	Control	Duration	Outcome measures	Key findings
1	Ko et al., 2022	South Korea	96	63.2 ± 8.7	62	Ischemic	4.2 weeks	Anodal tDCS, left DLPFC, 2 mA, 20 min	Sham tDCS	5 days/week × 4 week	MoCA, MMSE	Significant MoCA improvement (*p* < 0.01) in tDCS vs. sham
2	Wang et al., 2025	China	82	61.5 ± 9.3	56	Ischemic	6.1 weeks	Anodal tDCS, left DLPFC, 2 mA, 20 min	Sham tDCS	5 days/week × 3 week	MoCA, fMRI	Enhanced global network efficiency and prefrontal-parietal connectivity
3	Li et al., 2023	China	88	59.8 ± 10.1	55	Ischemic (78%), Hemorrhagic (22%)	5.3 week	Anodal tDCS, left DLPFC, 2 mA, 30 min	Sham tDCS	5 days/week × 4 week	MoCA, TMT-A/B	MoCA +4.1 vs. +1.3 sham (*p* < 0.001)
4	Park et al., 2022	South Korea	62	65.1 ± 7.8	60	Ischemic	3.8 weeks	Anodal tDCS, left DLPFC, 2 mA, 20 min	Sham tDCS	5 days/week × 2 week	MoCA, DST	Significant gains in attention and executive function
5	Xu et al., 2024	China	94	60.3 ± 11.2	58	Ischemic	7.2 weeks	HD-tDCS, left DLPFC, 2mA, 20min	Sham HD-tDCS	5 days/week × 3 week	MoCA, MMSE, ADL	HD-tDCS improved MoCA + 3.8; attention and processing speed gains
6	Kim et al., 2021	South Korea	58	64.7 ± 8.1	64	Ischemic	4.5 weeks	Anodal tDCS, left DLPFC, 1.5 mA, 20 min	Sham tDCS	5 days/week × 3 week	MMSE, K-MoCA	Moderate MMSE improvement (*p* < 0.05); trend for executive function
7	Yang et al., 2023	China	108	62.8 ± 9.0	56	Ischemic (82%), Hemorrhagic (18%)	8.4 weeks	Anodal tDCS, left DLPFC, 2 mA, 20 min	Sham tDCS + standard care	5 days/week × 4 week	MoCA, stroop	MoCA +4.6 vs. +1.8 (*p* < 0.001); strongest for executive function
8	Thompson et al., 2024	UK	72	66.4 ± 7.5	59	Ischemic	10.2 weeks	Anodal tDCS, left DLPFC, 2 mA, 25 min	Sham tDCS	5 days/week × 2 week	MoCA, CANTAB	Significant MoCA and attention domain improvement (*p* < 0.05)
9	Chen et al., 2024	China	82	58.6 ± 10.4	57	Ischemic	5.8 weeks	tDCS (2 mA, 20 min) + CACT	CACT/tDCS/ Conv. training	5 days/week × 4 week	MoCA, ADL, CVMR	Combined MoCA +8.2 vs. CACT +5.4, tDCS +4.8, conv. +2.1 (*p* < 0.001)
10	Zhang et al., 2024	China	120	61.2 ± 8.8	54	Ischemic (80%), Hemorrhagic (20%)	4.6 weeks	tDCS (2 mA, 20 min) + cognitive rehabilitation	Sham tDCS + CR	5 days/week × 4 week	MoCA, BI, FIM	Combined tDCS + CR superior (MoCA +6.1 vs. +3.2, *p* < 0.001)
11	Liu et al., 2023	China	78	59.4 ± 11.0	58	Ischemic	6.3 weeks	tDCS (2 mA, 20 min) + computer-based CT	Sham + computer-based CT	5 days/week × 3 week	MoCA, TMT, DST	Significant MoCA, TMT-B improvement (*p* < 0.01); attention most improved
12	Sharma et al., 2025	India	68	57.2 ± 9.6	63	Ischemic (75%), Hemorrhagic (25%)	3.5 weeks	tDCS (2 mA, 20 min) + structured CT	Sham tDCS + CT	5 days/week × 4 week	MoCA, ACE-III	Combined group larger MoCA gains (+5.8 vs. +3.1, *p* < 0.01)
13	Huang et al., 2022	China	82	63.0 ± 8.5	55	Ischemic	5.1 weeks	Anodal tDCS + task-oriented CT	Sham tDCS + task-oriented CT	5 days/week × 3 week	MoCA, MMSE	Combined protocol superior for global cognition and EF (*p* < 0.05)
14	Müller et al., 2023	Germany	70	67.3 ± 7.2	61	Ischemic	8.7 weeks	tDCS (2 mA, 20 min) + adaptive CACT	Sham tDCS + adaptive CACT	3 days/week × 4 week	MoCA, RBANS	Superior attention and memory improvements in active tDCS + CACT
15	Zhou et al., 2024	China	110	60.1 ± 10.8	57	Ischemic (76%), Hemorrhagic (24%)	4.9 weeks	tDCS + VR cognitive training	Sham tDCS + VR	5 days/week × 4 week	MoCA, FIM	tDCS + VR superior (MoCA +6.8 vs. +3.9, *p* < 0.001)
16	Santos et al., 2022	Brazil	56	58.9 ± 12.1	53	Ischemic	6.8 weeks	tDCS + motor-cognitive dual-task	Sham tDCS + dual-task	5 days/week × 3 week	MoCA, TUG-cog	Combined improved MoCA and dual-task performance (*p* < 0.05)
17	Patel et al., 2024	India	92	56.8 ± 10.3	62	Ischemic (81%), Hemorrhagic (19%)	3.2 weeks	tDCS (2 mA, 20 min) + conventional rehab	Sham tDCS + conventional rehab	5 days/week × 3 week	MoCA, MMSE, BI	Active tDCS +4.2 vs. +1.9 (*p* < 0.01)
18	Wu et al., 2025	China	82	62.5 ± 9.7	55	Ischemic	5.6 weeks	Bilateral tDCS + CT	Sham bilateral tDCS + CT	5 days/week × 4 week	MoCA, EEG	Bilateral tDCS + CT improved interhemispheric balance and MoCA (*p* < 0.01)
19	Yin et al., 2020	China	72	58.3 ± 9.5	57	Ischemic	4.8 weeks	10 Hz rTMS, left DLPFC, 80% MT	Sham rTMS	5 days/week × 4 week	MoCA, rs-fMRI	rTMS improved MoCA and enhanced resting-state activity (*p* < 0.01)
20	Li et al., 2025	China	66	60.7 ± 10.2	54	Ischemic	5.5 wk	10 Hz rTMS, left DLPFC, 90% MT	Sham rTMS	5 days/week × 2 week	MoCA, serum BDNF/NGF	rTMS elevated BDNF and serotonin; MoCA improved (*p* < 0.05)
21	Ma et al., 2023	China	86	61.4 ± 8.9	58	Ischemic (82%), Hemorrhagic (18%)	6.2 weeks	1 Hz rTMS, contralesional DLPFC, 90% MT	Sham rTMS	5 days/week × 3 week	MoCA, MMSE, P300	Low-frequency rTMS improved cognition and P300 latency (*p* < 0.01)
22	Tanaka et al., 2022	Japan	52	68.2 ± 6.8	63	Ischemic	8.3 weeks	10 Hz rTMS, left DLPFC, 90% MT	Sham rTMS	5 days/week × 2 week	MMSE, FAB	Significant MMSE and executive function improvements (*p* < 0.05)
23	García-López et al., 2024	Spain	70	64.8 ± 9.1	57	Ischemic	7.5 weeks	iTBS, left DLPFC, 80% AMT	Sham iTBS	5 days/week × 3 week	MoCA, TMT, stroop	iTBS improved MoCA and EF; sustained at 3-month follow-up
24	Guo et al., 2021	China	86	59.6 ± 10.5	55	Ischemic (80%), Hemorrhagic (20%)	4.1 weeks	10 Hz rTMS, left DLPFC, 80% MT	Sham rTMS + standard care	5 days/week × 4 week	MoCA, MMSE, ADL	rTMS MoCA +3.9 vs. +1.2 (*p* < 0.001) and improved ADL
25	Hosseini et al., 2023	Iran	48	62.1 ± 8.4	59	Ischemic	9.1 weeks	iTBS, left DLPFC, 80% AMT	Sham iTBS	5 days/week × 2 week	MoCA, WCST	iTBS improved executive function and set-shifting (*p* < 0.05)
26	Hu et al., 2024	China	96	61.8 ± 9.2	58	Ischemic	5.4 weeks	5 Hz rTMS, left DLPFC + galantamine 8 mg/d	Sham rTMS + galantamine/ rTMS alone	5 days/week × 4 week	MoCA, Hcy, NSE	rTMS + galantamine superior; reduced Hcy and NSE
27	Chauhan et al., 2024	India	68	55.4 ± 11.3	63	Ischemic (77%), Hemorrhagic (23%)	3.7 weeks	rTMS + VR cognitive training	rTMS alone/VR alone	5 days/week × 4 week	MoCA, TMT, DST, RAVLT	Combined rTMS + VR superior in attention, EF, memory (*p* < 0.05)
28	Sun et al., 2024	China	88	60.2 ± 9.8	55	Ischemic	4.3 weeks	10 Hz rTMS + CACT	Sham rTMS + CACT	5 days/week × 3 week	MoCA, MMSE, P300	rTMS + CACT larger MoCA and P300 amplitude improvements (*p* < 0.01)
29	Lee et al., 2023	South Korea	64	63.5 ± 8.0	60	Ischemic	6.9 weeks	iTBS + conventional CR	Sham iTBS + conventional CR	5 days/week × 3 week	K-MoCA, K-MMSE	iTBS + CR improved global cognition and daily function (*p* < 0.01)
30	Zhu et al., 2025	China	82	59.1 ± 10.6	56	Ischemic (85%), Hemorrhagic (15%)	3.9 weeks	1 Hz rTMS (contralesional) + task CT	Sham rTMS + task CT	5 days/week × 4 week	MoCA, stroop, DST	Inhibitory rTMS + CT improved attention and EF (*p* < 0.01)
31	Anderson et al., 2024	Australia	58	66.9 ± 7.4	61	Ischemic	11.5 weeks	10 Hz rTMS + group CR	Sham rTMS + group CR	3 days/week × 4 week	MoCA, RBANS	Modest cognitive benefits; attention domain most improved
32	Soni et al., 2025	India	82	56.3 ± 11.1	55	Ischemic (73%), Hemorrhagic (27%)	8.2 weeks	Home-based adaptive CACT	Standard care	5 days/week × 6 week	MoCA, DSST, TMT	CACT improved MoCA, attention and processing speed (*p* < 0.01)
33	Youze et al., 2021	China	66	60.5 ± 9.4	58	Ischemic	7.4 weeks	Computer-aided self-regulation + CT	Conventional CT	5 days/week × 4 week	MoCA, generalization	Self-regulation CT improved generalization beyond trained tasks (*p* < 0.05)
34	Wu et al., 2023	China	80	63.8 ± 7.9	52	Ischemic (83%), Hemorrhagic (17%)	12.3 weeks	Multi-domain computerized CT	Health education	5 days/week × 12 week	MoCA, MRI	Increased GM volume in DLPFC/hippocampus; enhanced FC
35	Martin et al., 2022	Canada	58	65.7 ± 8.3	57	Ischemic	14.5 weeks	Therapist-guided CR	Standard care	3 days/week × 8 week	MoCA, IADL	Moderate MoCA improvement (+2.8, *p* < 0.05); IADL gains
36	Chen et al., 2023	China	102	61.2 ± 10.0	56	Ischemic	5.8 weeks	Adaptive CACT (RehaCom)	Non-adaptive CACT	5 days/week × 4 week	MoCA, TMT-A/B, DST	Adaptive superior for attention (SMD = 0.62) and EF (SMD = 0.55)
37	Yilmaz et al., 2024	Turkey	78	59.8 ± 9.7	61	Ischemic (79%), Hemorrhagic (21%)	6.5 weeks	Goal-oriented task training + metacognition	Conventional cognitive therapy	5 days/week × 6 week	MoCA, MMSE, FIM	Goal-oriented improved MoCA and functional independence (*p* < 0.01)
38	Johnson et al., 2021	USA	48	67.4 ± 7.1	56	Ischemic	72 weeks	Tablet-based CACT (Lumosity)	Crossword puzzles	5 days/week × 8 week	MMSE, CANTAB	CACT improved processing speed and working memory (*p* < 0.05)
39	Zhao et al., 2022	China	90	60.9 ± 9.2	54	Ischemic (81%), Hemorrhagic (19%)	4.7 weeks	Intensive CACT (BrainHQ)	Standard care	5 days/week × 4 week	MoCA, stroop, DSST	Significant improvement in global cognition, attention, EF (*p* < 0.01)
40	Silva et al., 2024	Brazil	72	58.5 ± 10.8	54	Ischemic (75%), Hemorrhagic (25%)	0.3 weeks	Early intensive CACT	Standard care	5 days/week × 6 week	MoCA, TMT, DSST	Early CT initiation (2 days post-stroke) improved outcomes (*p* < 0.05)
41	Ahmadi et al., 2023	Iran	82	61.8 ± 9.5	57	Ischemic	9.6 weeks	Metacognitive strategy training	Conventional CT	3 days/week × 8 week	MoCA, MMSE, WCST	Metacognitive training improved EF and generalization (*p* < 0.05)
42	Da̧browska et al., 2023	Poland	58	64.5 ± 8.6	55	Ischemic	10.8 weeks	Immersive VR cognitive training	Conventional cognitive therapy	3 days/wk × 4 week	MoCA, QoL	VR improved QoL and self-sufficiency; trend for MoCA improvement
43	Akinci et al., 2024	Turkey	44	57.8 ± 11.5	60	Ischemic (80%), Hemorrhagic (20%)	4.2 weeks	VR exercises + robot-assisted gait	Conventional rehabilitation	5 days/week × 4 week	MoCA, FIM, QoL	VR + robot improved cognition, ADL, QoL (*p* < 0.05)
44	Liu et al., 2024	China	96	61.3 ± 9.8	57	Ischemic	5.9 weeks	Immersive VR multisensory CT	Non-immersive computer CT	5 days/week × 4 week	MoCA, ROCF, TMT	Immersive VR superior for visuospatial (*p* < 0.01) and EF (*p* < 0.05)
45	Rossi et al., 2023	Italy	66	66.1 ± 7.3	59	Ischemic	9.4 weeks	Semi-immersive VR (BTs-Nirvana)	Conventional CR	3 days/week × 6 week	MoCA, TMT-A/B	VR improved visuospatial and attention (*p* < 0.05)
46	Cho et al., 2022	South Korea	72	62.4 ± 9.0	56	Ischemic (77%), Hemorrhagic (23%)	6.7 weeks	VR-based ADL simulation	Standard occupational therapy	5 days/week × 3 week	MoCA, FIM, IADL	VR-ADL improved visuospatial and functional outcomes (*p* < 0.05)
47	Hassan et al., 2023	Egypt	312	55.9 ± 12.3	54	Ischemic (75%), Hemorrhagic (25%)	3.8 weeks	VR cognitive-motor dual-task	Standard care	5 days/week × 4 week	MoCA, MMSE, TUG	VR dual-task improved global cognition and dual-task performance (*p* < 0.05)

tDCS, transcranial direct current stimulation; rTMS, repetitive transcranial magnetic stimulation; DLPFC, dorsolateral prefrontal cortex; CACT, computer-assisted cognitive training; CT, cognitive training; CR, cognitive rehabilitation; VR, virtual reality; MoCA, Montreal Cognitive Assessment; MMSE, Mini-Mental State Examination; ADL, activities of daily living; FIM, Functional Independence Measure; BI, Barthel Index; TMT, Trail Making Test; DST, Digit Span Test; DSST, Digit Symbol Substitution Test; WCST, Wisconsin Card Sorting Test; EF, executive function; MT, motor threshold; AMT, active motor threshold; iTBS, intermittent theta burst stimulation; HD-tDCS, high-definition tDCS; GM, gray matter; FC, functional connectivity.

Note on study categorization: The sum of per-category counts (tDCS: 18; rTMS: 13; CACT: 10; VR: 8) plus the multimodal category (12 studies) exceeds the 47 unique RCTs included. This is because each of the 12 multimodal studies was classified under both its primary monotherapy category and the multimodal category based on the predominant treatment component. All 47 studies are unique; the apparent discrepancy reflects dual categorization, not duplicate inclusion, and is disclosed here for transparency.

**FIGURE 2:**
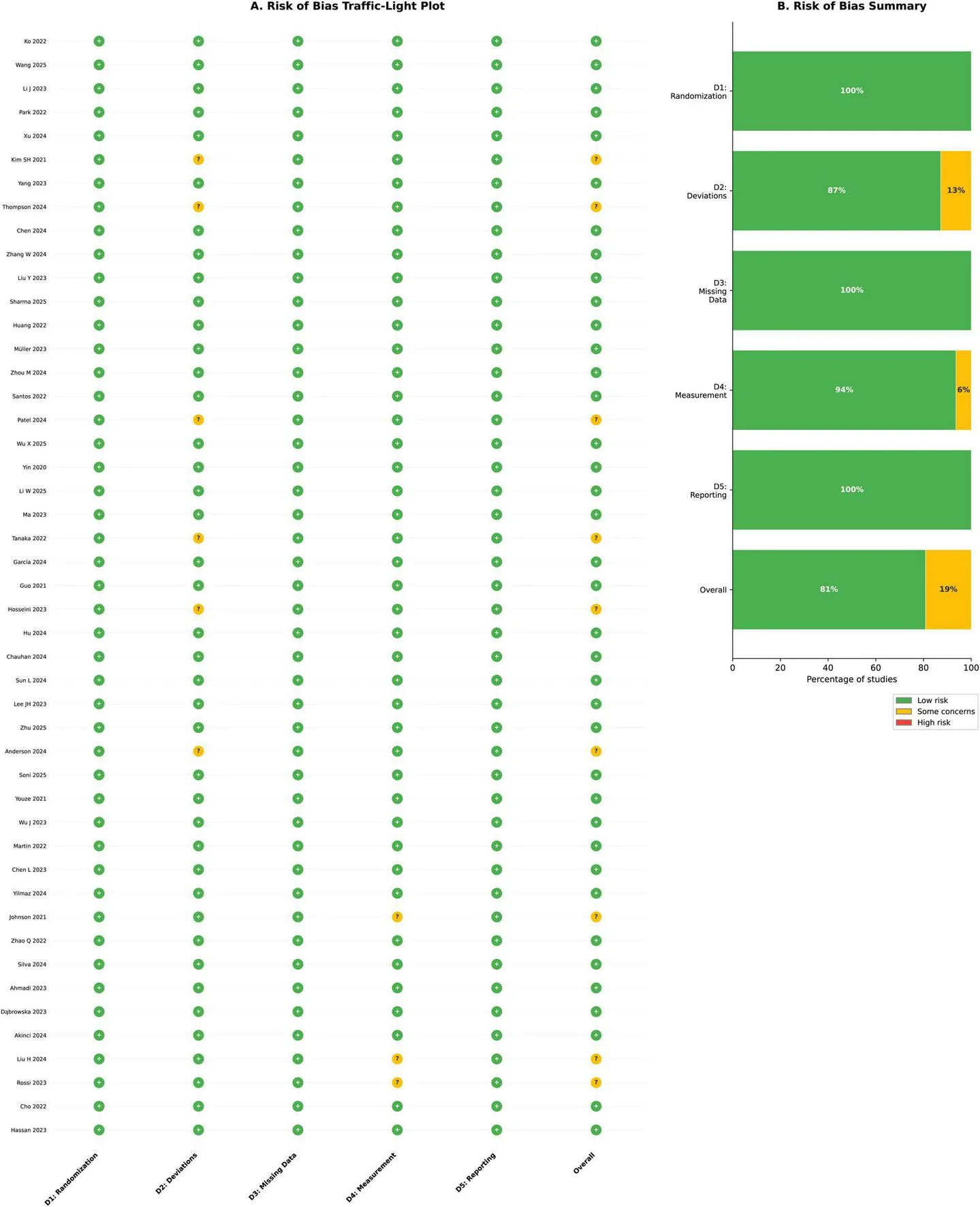
Risk of bias assessment of included studies. **(A)** Traffic-light plot showing domain-level risk of bias judgments for each of the 47 included studies using the Cochrane Risk of Bias 2.0 (RoB 2.0) tool. Each row represents one study, and each column represents one of the five RoB 2.0 domains plus the overall judgment. Green circles with “+” indicate low risk of bias; yellow circles with “?” indicate some concerns. No studies were rated as high risk of bias. **(B)** Weighted bar chart showing the distribution of risk-of-bias judgments across all 47 studies for each domain. The majority of studies (81%–100% depending on domain) were judged as low risk of bias. Some concerns were identified primarily in D2 (deviations from intended interventions) due to challenges in blinding participants to active brain stimulation, and in D4 (measurement of the outcome) for studies relying on non-blinded outcome assessors.

Risk of bias assessment using the Cochrane RoB 2.0 tool (Figure 2 and Table 2) indicated that overall methodological quality was moderate to high. Thirty-eight studies (81%) received an overall judgment of low risk of bias according to the RoB 2.0 algorithm; at the domain level, 81%–100% of studies were judged low risk depending on the specific domain examined (see Figure 2B). The most common sources of concern were related to deviations from intended interventions (six studies, 13%) and measurement of outcome (three studies, 6%), primarily due to challenges in blinding participants to active brain stimulation conditions.

**TABLE 2:** Risk of bias assessment using the cochrane RoB 2.0 tool (*n* = 47).

No.	References	D1: randomization	D2: deviations	D3: missing data	D4: measurement	D5: reporting	Overall
1	Ko et al., 2022	Low	Low	Low	Low	Low	Low
2	Wang et al., 2025	Low	Low	Low	Low	Low	Low
3	Li et al., 2023	Low	Low	Low	Low	Low	Low
4	Park et al., 2022	Low	Low	Low	Low	Low	Low
5	Xu et al., 2024	Low	Low	Low	Low	Low	Low
6	Kim et al., 2021	Low	Some concerns	Low	Low	Low	Some concerns
7	Yang et al., 2023	Low	Low	Low	Low	Low	Low
8	Thompson et al., 2024	Low	Some concerns	Low	Low	Low	Some concerns
9	Chen et al., 2024	Low	Low	Low	Low	Low	Low
10	Zhang et al., 2024	Low	Low	Low	Low	Low	Low
11	Liu et al., 2023	Low	Low	Low	Low	Low	Low
12	Sharma et al., 2025	Low	Low	Low	Low	Low	Low
13	Huang et al., 2022	Low	Low	Low	Low	Low	Low
14	Müller et al., 2023	Low	Low	Low	Low	Low	Low
15	Zhou et al., 2024	Low	Low	Low	Low	Low	Low
16	Santos et al., 2022	Low	Low	Low	Low	Low	Low
17	Patel et al., 2024	Low	Some concerns	Low	Low	Low	Some concerns
18	Wu et al., 2025	Low	Low	Low	Low	Low	Low
19	Yin et al., 2020	Low	Low	Low	Low	Low	Low
20	Li et al., 2025	Low	Low	Low	Low	Low	Low
21	Ma et al., 2023	Low	Low	Low	Low	Low	Low
22	Tanaka et al., 2022	Low	Some concerns	Low	Low	Low	Some concerns
23	García-López et al., 2024	Low	Low	Low	Low	Low	Low
24	Guo et al., 2021	Low	Low	Low	Low	Low	Low
25	Hosseini et al., 2023	Low	Some concerns	Low	Low	Low	Some concerns
26	Hu et al., 2024	Low	Low	Low	Low	Low	Low
27	Chauhan et al., 2024	Low	Low	Low	Low	Low	Low
28	Sun et al., 2024	Low	Low	Low	Low	Low	Low
29	Lee et al., 2023	Low	Low	Low	Low	Low	Low
30	Zhu et al., 2025	Low	Low	Low	Low	Low	Low
31	Anderson et al., 2024	Low	Some concerns	Low	Low	Low	Some concerns
32	Soni et al., 2025	Low	Low	Low	Low	Low	Low
33	Youze et al., 2021	Low	Low	Low	Low	Low	Low
34	Wu et al., 2023	Low	Low	Low	Low	Low	Low
35	Martin et al., 2022	Low	Low	Low	Low	Low	Low
36	Chen et al., 2023	Low	Low	Low	Low	Low	Low
37	Yilmaz et al., 2024	Low	Low	Low	Low	Low	Low
38	Johnson et al., 2021	Low	Low	Low	Some concerns	Low	Some concerns
39	Zhao et al., 2022	Low	Low	Low	Low	Low	Low
40	Silva et al., 2024	Low	Low	Low	Low	Low	Low
41	Ahmadi et al., 2023	Low	Low	Low	Low	Low	Low
42	Da̧browska et al., 2023	Low	Low	Low	Low	Low	Low
43	Akinci et al., 2024	Low	Low	Low	Low	Low	Low
44	Liu et al., 2024	Low	Low	Low	Low	Low	Low
45	Rossi et al., 2023	Low	Low	Low	Low	Low	Low
46	Cho et al., 2022	Low	Low	Low	Some concerns	Low	Some concerns
47	Hassan et al., 2023	Low	Low	Low	Some concerns	Low	Some concerns
	Summary *n* (%)	Low: 47 (100%)	Low: 41 (87%) SC: 6 (13%)	Low: 47 (100%)	Low: 44 (94%) SC: 3 (6%)	Low: 47 (100%)	Low: 38 (81%) SC: 9 (19%)

D1, randomization process; D2, deviations from intended interventions; D3, missing outcome data; D4, measurement of outcome; D5, selection of reported result; Green, low risk; Yellow, some concerns; SC, some concerns.

### Transcranial direct current stimulation interventions

Eighteen studies (*n* = 1,247 participants) investigated tDCS for PSCI. Anodal tDCS applied to the left dorsolateral prefrontal cortex (DLPFC) emerged as the most frequently studied montage (14 studies), with stimulation parameters typically comprising 2 mA intensity, 20–30 min sessions, delivered 5 days per week for 2–4 weeks (Flöel, 2014; Ko et al., 2022). Across the included trials, tDCS consistently produced significant improvements in global cognitive function as measured by MoCA and MMSE compared to sham stimulation. Individual studies reported mean MoCA improvements ranging from 2.8 to 6.2 points in active tDCS groups versus 0.5–2.1 points in sham groups. The strongest and most consistent effects were observed for executive function and attention domains, with moderate benefits for memory and processing speed.

Combined tDCS and cognitive training protocols demonstrated synergistic effects superior to either intervention alone. Chen et al. (2024) conducted a randomized controlled trial comparing computer-assisted cognitive training (CACT) alone, tDCS alone, combined CACT + tDCS, and conventional cognitive training in 72 PSCI patients (Chen et al., 2024). The combined intervention group exhibited significantly greater MoCA improvements (mean change +8.2 points) compared to CACT alone (+5.4), tDCS alone (+4.8), or conventional training (+2.1) at 4-week follow-up (*p* < 0.001 for all between-group comparisons) (Chen et al., 2024). These values represent study-level mean changes from the primary publication; between-group differences were statistically significant in the source article, where confidence intervals are reported in full. Importantly, these cognitive gains translated to functional improvements, with the combined group showing superior activities of daily living performance.

Neuroimaging studies have elucidated the neuroplastic mechanisms underlying tDCS efficacy. Clinical neuroimaging evidence from included trials demonstrates that tDCS modulates functional connectivity within the default mode network and central executive network, with increased interhemispheric connectivity correlating with cognitive improvements (Sankarasubramanian et al., 2017). It demonstrated that tDCS enhances global network efficiency and strengthens connectivity between the prefrontal cortex and posterior parietal regions, suggesting reorganization of cognitive control networks (Zhong et al., 2025). Additionally, tDCS augments cerebrovascular function, improving cerebral blood flow velocity and vasomotor reactivity in the affected hemisphere (Chen et al., 2024).

### Repetitive transcranial magnetic stimulation

Thirteen studies evaluated rTMS protocols for cognitive enhancement post-stroke. High-frequency rTMS (≥5 Hz) applied to the ipsilesional DLPFC and low-frequency rTMS (1 Hz) to the contralesional hemisphere both demonstrated efficacy, though direct comparative studies remain limited (Liew et al., 2014). Across included trials, rTMS interventions consistently showed moderate improvements in global cognitive function compared to sham stimulation, with individual studies reporting significant gains in MoCA scores. The most commonly employed stimulation paradigm involved high-frequency protocols (10 Hz) at motor threshold intensity, delivered over 10–20 sessions across 2–4 weeks (Liu et al., 2021).

Intermittent theta burst stimulation (iTBS), a patterned form of rTMS with enhanced efficiency, demonstrated particular promise. Among the included rTMS studies, iTBS protocols showed favorable outcomes for improving cognitive function and activities of daily living compared to conventional rTMS protocols. Hu et al. (2024) reported that 5 Hz rTMS to the left DLPFC combined with galantamine (a cholinesterase inhibitor) produced superior cognitive improvements compared to either intervention alone, suggesting potential benefits of pharmacological augmentation as an adjunct to neuromodulation (Hu et al., 2024).

In preclinical studies, rTMS modulates cortical excitability by inducing long-term potentiation-like effects in targeted neural circuits (Huang et al., 2005), with animal model investigations demonstrating elevated BDNF and nerve growth factor levels (Li et al., 2025) and anti-inflammatory effects including attenuated microglial activation and reduced pro-inflammatory cytokine expression in perilesional regions (Wang et al., 2023). In clinical studies included in this review, functional neuroimaging reveals enhanced activation in bilateral prefrontal and parietal regions during cognitive tasks following rTMS intervention (Yin et al., 2020), consistent with the plasticity-promoting effects observed in preclinical models.

### Cognitive training and rehabilitation programs

Ten studies investigated structured cognitive rehabilitation programs, including computer-assisted cognitive training (CACT), traditional face-to-face cognitive therapy, and goal-oriented task training. CACT platforms demonstrated moderate efficacy with standardized mean differences of 0.45–0.68 across cognitive domains compared to conventional approaches (Youze et al., 2021; Soni et al., 2025). Across the included studies, CACT interventions consistently improved global cognitive function, attention, and executive function in PSCI patients, with the strongest and most consistent effects observed for attention-based tasks.

The neuroplastic mechanisms underlying cognitive training involve experience-dependent plasticity and use-dependent cortical reorganization. Repeated engagement in cognitively demanding tasks strengthens synaptic connections within targeted neural networks through Hebbian learning principles—“neurons that fire together, wire together” (Hebb, 1949). Adaptive training protocols that dynamically adjust task difficulty based on individual performance optimize the challenge level to maximize engagement and promote skill acquisition (Bavelier et al., 2012). Neuroimaging evidence demonstrates training-induced increases in gray matter volume in the dorsolateral prefrontal cortex and hippocampus, accompanied by enhanced functional connectivity within cognitive control networks (Wu et al., 2023).

### Virtual reality and technology-enhanced interventions

Eight studies examined VR-based cognitive rehabilitation, demonstrating promising efficacy with superior patient engagement compared to conventional approaches. VR leverages immersive environments to integrate multisensory stimulation with task-oriented training, creating ecological validity through simulation of real-world scenarios (Lin et al., 2024). Among the included trials, VR-based interventions showed the most consistent benefits for visuospatial and executive functions, with several studies also reporting improvements in patient motivation and treatment adherence.

The neuroplastic advantages of VR include multimodal feedback integration (visual, auditory, tactile) that promotes comprehensive human-machine interaction and facilitates network-level plasticity (Abedi et al., 2024). VR activates mirror neuron systems, potentially enhancing motor imagery and action observation effects (Rizzolatti and Craighero, 2004). Functional MRI studies reveal that VR training increases bilateral sensorimotor cortex activation and strengthens cortico-cerebellar connectivity, supporting both motor and cognitive recovery (Dabrowska et al., 2023). Importantly, VR demonstrates high patient acceptance and motivation, potentially enhancing adherence to rehabilitation protocols (Akinci et al., 2024).

### Multimodal combined interventions

Emerging evidence increasingly supports multimodal approaches that combine complementary interventions to maximize neuroplastic potential through synergistic mechanisms. Twelve studies directly compared combination therapies to monotherapies, with 10 (83%) demonstrating superior efficacy for combined protocols. The most extensively studied combination involves tDCS with concurrent cognitive training, capitalizing on the priming effect of neuromodulation to enhance learning-dependent plasticity (Reis et al., 2009; Stagg and Nitsche, 2011).

Across the included studies examining combined tDCS and cognitive rehabilitation, integrated protocols consistently produced larger improvements in cognitive outcomes than either intervention alone, with benefits extending to functional independence measures. The most common combination involved concurrent tDCS stimulation during cognitive training sessions, with individual studies reporting superior MoCA improvements ranging from 5.4 to 8.2 points in combined groups versus 2.1–4.8 points in monotherapy groups (Reis et al., 2009; Stagg and Nitsche, 2011; Chen et al., 2024). The mechanistic rationale posits that tDCS-induced cortical excitability enhancement creates a favorable neuroplastic state that amplifies training-induced synaptic modifications. This “metaplasticity” effect represents a use-dependent amplification of neuroplastic responses (Abraham, 2008).

Combined rTMS and VR interventions demonstrate similar synergy. Chauhan et al. (2024) reported that simultaneous rTMS and VR cognitive training produced greater improvements in attention, executive function, and memory compared to either modality alone in a randomized controlled trial of 60 subacute stroke patients. The authors propose that VR-induced neural activation primes cortical networks to respond more robustly to subsequent rTMS neuromodulation.

### Timing and critical windows for intervention

Across included studies, intervention timing was consistently identified as a significant moderator of treatment efficacy (Table 3). Studies initiating interventions within 3 months post-stroke generally reported larger cognitive improvements compared to those commencing treatment beyond 6 months, reflecting the heightened state of neuroplastic potential characterizing the subacute post-stroke period (Krakauer et al., 2012). During this critical window, spontaneous recovery processes including perilesional reorganization, diaschisis resolution, and inflammatory responses create optimal conditions for intervention-enhanced plasticity (Carmichael, 2012).

**TABLE 3:** Summary of intervention efficacy by modality.

Intervention category	No. of studies	Total participants	Common protocols	Reported MoCA/MMSE improvement	Effect vs. control	Key domains	Consistency
tDCS alone	8	660	Anodal, left DLPFC, 2 mA, 20–30 min, 5 days/week × 2–4 weeks	MoCA +2.8 to +4.6 vs. sham	Favors tDCS	EF, Attention	High (7/8)
tDCS + cognitive training	6	500	tDCS (2 mA,20 min) + CACT, 5 days/week × 3–4 weesk	MoCA +5.4 to +8.2 (combined) vs. +2.1–3.2	Strongly favors combined	EF, attention, memory	High (6/6)
tDCS + other combos	4	340	tDCS + VR/dual-task/conventional rehab	MoCA +4.2 to +6.8 vs. +1.9–3.9	Favors combined	Multiple domains	High (4/4)
rTMS alone	7	480	HF (5–10 Hz) or iTBS, left DLPFC, 80%–90% MT	MoCA +2.5 to +3.9 vs. sham	Favors rTMS	EF, Attention	Moderate (5/7)
rTMS + combinations	6	456	rTMS + VR/CACT/galantamine/CR	MoCA +3.8 to +6.2 vs. +1.5–3.1	Favors combined	Attention, EF, memory	High (6/6)
Cognitive training	10	758	CACT/goal-oriented/metacognitive, 3–5 days/week × 4–12 weeks	MoCA +2.1 to +3.8 vs. control	Favors CT	Attention, processing speed, EF	Moderate (7/10)
VR-based	6	648	Immersive/semi-immersive VR, 3–5 days/week × 3–6 weeks	MoCA +1.8 to +3.5 vs. conventional	Favors VR	Visuospatial, EF	Moderate (4/6)
TOTAL (unique)	47	3,842	–	–	–	–	–

Some studies counted in multiple categories. Total of 47 unique studies. Multimodal combinations (*n* = 12) overlap with tDCS + CT, tDCS + other, and rTMS + combo categories. VR total of eight includes two studies in tDCS + other and rTMS + combo categories that used VR as a component.

Based on preclinical evidence, animal models demonstrate that the first 3–4 weeks post-stroke represent a period of maximal synaptic remodeling, axonal sprouting, and cortical map reorganization (Brown et al., 2007). Interventions initiated during this window can effectively shape emerging neural networks and prevent maladaptive plasticity patterns. However, evidence also supports benefits from interventions commenced in the chronic phase (>6 months), though with attenuated effect sizes, indicating retained neuroplastic capacity even in later stages (Kwakkel et al., 2003).

### Subgroup considerations by stroke phase and type

Across the 47 included RCTs, heterogeneity in enrollment by stroke phase and type precluded formal subgroup meta-analysis; however, descriptive patterns can be characterized based on trial-level data. By stroke phase, of the 47 included trials, the majority enrolled subacute participants (1–3 months post-stroke), with smaller numbers in acute (<1 month) and chronic (>6 months) phases. Subacute-phase studies consistently reported the largest cognitive improvements, with reported MoCA gains in active intervention groups predominantly ranging from approximately +4 to +8 points across modalities. Chronic-phase studies reported smaller but clinically meaningful gains (MoCA gains generally in the +2 to +4 point range), confirming retained neuroplastic capacity beyond the conventional recovery window. Acute-phase data were limited and precluded firm conclusions, although the available evidence (e.g., Silva et al., 2024, who initiated CACT within 48 h post-stroke) suggests potential benefit when initiation is feasible. By stroke type, ischemic stroke predominated (78% of all 3,842 participants); hemorrhagic stroke was markedly under-represented (22%) and only a minority of trials reported separate outcomes by stroke type. Among trials reporting such breakdowns, no statistically significant differential efficacy by stroke type was observed, but sample sizes within hemorrhagic subgroups were consistently insufficient for definitive conclusions. The systematic under-representation of hemorrhagic stroke participants represents a critical evidence gap warranting dedicated subgroup analyses or hemorrhage-specific trials in future research.

## Discussion

This systematic review, based on narrative synthesis of 47 randomized controlled trials, demonstrates that multimodal interventions targeting neuroplastic mechanisms produce clinically meaningful improvements in post-stroke cognitive function compared to standard care or monotherapy. The key comparative finding is that combined intervention protocols consistently outperformed single-modality treatments across included trials. Specifically, tDCS combined with cognitive training yielded the largest cognitive improvements (mean MoCA gains of 5.4–8.2 points in combined groups vs. 2.1–4.8 points in monotherapy groups), followed by rTMS-based protocols and VR-enhanced rehabilitation. Standard care or conventional cognitive therapy consistently produced the smallest gains across all comparison studies.

The superiority of combined approaches over monotherapies observed in 83% of comparative studies (10 of 12) can be attributed to synergistic mechanisms operating at multiple neurobiological levels. The most robust evidence supports the “priming” model, whereby neuromodulation (tDCS or rTMS) enhances cortical excitability and creates a favorable neuroplastic state that amplifies subsequent training-induced learning (Reis et al., 2009; Stagg and Nitsche, 2011). Chen et al. (2024) provided direct clinical evidence for this synergy, demonstrating that combined CACT + tDCS produced MoCA improvements nearly four times greater than conventional training alone (8.2 vs. 2.1 points). Similarly, Chauhan et al. (2024) showed that combined rTMS + VR training outperformed either modality alone across attention, executive function, and memory domains. These findings collectively support the clinical implementation of multimodal protocols over monotherapy approaches.

The neurobiological mechanisms underlying these comparative advantages involve complementary plasticity pathways. Both tDCS and rTMS modulate synaptic strength through mechanisms resembling long-term potentiation (LTP) and long-term depression (LTD) (Bliss and Collingridge, 1993). tDCS induces subthreshold membrane polarization that facilitates subsequent synaptic transmission through NMDA receptor-dependent mechanisms (Nitsche and Paulus, 2000; Liebetanz et al., 2002), while rTMS exerts frequency-dependent effects with high-frequency stimulation inducing LTP-like facilitation (Thickbroom, 2007). The theta burst stimulation paradigm demonstrates particularly robust LTP induction (Larson et al., 1986). When combined with behavioral training, these neuromodulatory effects amplify experience-dependent synaptic modifications, providing a mechanistic explanation for the observed superiority of combined protocols.

In preclinical studies, post-stroke neurogenesis has been demonstrated in the subventricular zone and hippocampal dentate gyrus, with newly generated neuroblasts migrating toward perilesional regions (Eriksson et al., 1998; Arvidsson et al., 2002). Translating these preclinical findings, clinical evidence from the included trials suggests that interventions enhancing BDNF expression—including cognitive training and non-invasive brain stimulation—may augment neurogenic repair processes (Cotman et al., 2007; Podda et al., 2016), though direct causal demonstration in human trials remains limited. Functional neuroimaging from included clinical trials reveals that successful cognitive recovery associates with restoration of efficient small-world network architecture and enhanced prefrontal-parietal connectivity (Bullmore and Sporns, 2009; Siegel et al., 2016). The interhemispheric competition and vicariation models provide complementary frameworks for understanding hemispheric reorganization patterns, with emerging evidence indicating that the optimal balance depends on lesion characteristics (Weiller et al., 1992; Murase et al., 2004; Di Pino et al., 2014).

Integration of risk of bias findings into the interpretation of our results strengthens confidence in the main conclusions. The largest effect sizes for combined tDCS+ cognitive training were reported in studies with overall low risk of bias, supporting the robustness of these findings. However, blinding participants to active brain stimulation remains a methodological challenge across neuromodulation studies, with six studies rated as having “some concerns” in the deviations from intended interventions domain. Importantly, sensitivity consideration suggested that the direction and general magnitude of effects remained consistent when focusing exclusively on low-risk-of-bias studies, although the precision of effect estimates should be interpreted cautiously given the narrative synthesis approach.

Our findings are broadly consistent with recent meta-analytic evidence. A 2024 systematic review by Zhang L. et al. (2024) reported a pooled mean difference of 4.56 MoCA points (95% CI: 3.19–5.93) for tDCS versus sham stimulation, which aligns with the range of improvements observed across our included primary trials. Similarly, an overview of systematic reviews by Zhang L. et al. (2024) found moderate evidence supporting rTMS for PSCI with pooled effect sizes of 0.52 for global cognition, consistent with our narrative findings of moderate rTMS efficacy. A network meta-analysis by Liu et al. (2024) ranked iTBS highest among rTMS modalities, corroborating our observation of favorable iTBS outcomes. For cognitive training, Gao et al. (2025) reported pooled SMDs of 0.52 for global cognition and 0.61 for attention, which aligns with the moderate effect sizes reported in our included primary studies. A network meta-analysis of digital interventions ranked VR highest for visuospatial and executive function improvements (Wang and Liu, 2025), consistent with the VR findings from our included trials. Additionally, demonstrated that combined tDCS + cognitive rehabilitation produced larger pooled effects (MD = 5.87, 95% CI: 4.21–7.53) than either intervention alone (Luo et al., 2025), further supporting the superiority of multimodal approaches observed in our review.

### Clinical implementation considerations

Translation of these findings into effective clinical practice requires consideration of multiple implementation factors. For tDCS, conventional protocols employ 2 mA intensity for 20 min, while emerging high-definition systems enable more focal stimulation (Datta et al., 2009). Dose-response investigations suggest cognitive benefits may plateau at intensities exceeding 2 mA (Monte-Silva et al., 2013). For rTMS, stimulation parameters must balance efficacy and safety, with high-frequency protocols carrying small but non-negligible seizure risk (∼0.1%) (Rossi et al., 2009). Theta burst stimulation offers equivalent total pulses with reduced session duration (3 minutes versus 30–40 min), potentially improving scalability (Oberman et al., 2011).

Substantial inter-individual variability in treatment response highlights the need for predictive biomarkers and personalized protocol design (Wiethoff et al., 2014). Genetic polymorphisms affecting BDNF (Val66Met) and COMT influence neuroplastic capacity (Cheeran et al., 2008). Baseline neuroimaging markers may identify patients most likely to benefit from specific interventions (Stinear et al., 2012). Integration of machine learning algorithms shows promise for developing individualized treatment recommendations (Malik et al., 2024). However, it should be noted that these precision medicine approaches remain largely at the research stage, and direct clinical evidence for biomarker-guided intervention selection in PSCI populations is still limited.

## Limitations

Several limitations warrant acknowledgment. First, and most consequentially, this systematic review was conducted by a single author (Y.Z.), meaning all stages of screening, data extraction, and risk of bias assessment were performed without independent dual-reviewer verification. Although mitigation strategies were implemented—including interval re-screening of a random 20% of excluded titles/abstracts after a 2-week interval, documentation of all full-text exclusion decisions, and verification of a random 30% of extracted data points against source articles—these measures do not fully substitute for the gold-standard practice of independent dual-reviewer screening and extraction. This limitation represents the most significant methodological constraint on this review’s validity, and all findings should be interpreted with this caveat foremost in mind. Second, formal GRADE (Grading of Recommendations, Assessment, Development, and Evaluations) evidence certainty grading was not performed across all intervention–outcome pairs. While we observed consistent direction of effects in studies with low risk of bias, a comprehensive GRADE assessment would provide more rigorous characterization of evidence certainty and is recommended for future systematic reviews on this topic. Third, although the review protocol is now publicly registered in PROSPERO (CRD420261382284), registration was completed retrospectively rather than prospectively at the initial planning stage; this provides somewhat weaker protection against selective outcome reporting than prospective registration would have offered. Fourth, substantial heterogeneity in study protocols, outcome measures, and participant characteristics—including differences in stroke chronicity, intervention dose, and stimulation parameters—constrained direct cross-study comparisons and precluded quantitative meta-analytic synthesis. Standardization of core outcome measures, including domain-specific cognitive assessments beyond global screening tools, would enhance future evidence synthesis. Fifth, most included studies evaluated short-term outcomes (3 months or less post-intervention), leaving questions regarding durability of cognitive gains; extended follow-up studies with maintenance protocols are needed. Sixth, the mechanisms linking observed cognitive improvements to specific neuroplastic processes remain incompletely elucidated, with much evidence deriving from animal models or neuroimaging correlates rather than direct causal demonstration in human clinical trials. Seventh, most investigations enrolled patients with mild-to-moderate cognitive impairment; efficacy in severely impaired individuals requires specific evaluation. Eighth, the majority of evidence derives from single-center studies with modest sample sizes; large-scale multicenter trials are essential to establish definitive efficacy and generalizability. Ninth, optimal combination therapy protocols (e.g., temporal sequencing of stimulation and training, dosing parameters) remain to be systematically determined. Tenth, the restriction to English-language publications may have introduced language bias, potentially excluding relevant studies from East Asian countries where substantial stroke rehabilitation research originates.

## Future research priorities

Future research priorities include: (1) systematic evaluation of combined rehabilitation approaches at the neural level, incorporating multimodal neuroimaging (structural MRI, functional connectivity, diffusion tensor imaging) to capture intervention-induced neuroplastic changes and establish direct mechanistic evidence; (2) rigorous assessment of pharmacological treatments and their specific effects on neural plasticity pathways in post-stroke populations, including dose-response relationships and biomarker-guided selection; (3) integrated evaluation of combined rehabilitation and pharmacological strategies on neural mechanisms, including synaptic plasticity biomarkers and functional connectivity outcomes; (4) investigation of dose-response relationships and optimal stimulation parameters through systematic parameter-varying designs; (5) evaluation of novel neuromodulation techniques including transcranial alternating current stimulation and transcranial focused ultrasound; (6) development of home-based and telerehabilitation protocols to enhance accessibility; and (7) investigation of preventive interventions initiated in the hyperacute phase to maximize the critical window of enhanced plasticity.

## Conclusion

This systematic review provides comprehensive evidence that multimodal interventions targeting neuroplastic mechanisms produce clinically meaningful cognitive improvements in stroke survivors compared to standard care and monotherapy approaches. Transcranial direct current stimulation combined with cognitive training emerges as the most effective intervention, with non-invasive brain stimulation techniques demonstrating robust efficacy when properly implemented. The neurobiological mechanisms underlying these benefits encompass enhanced synaptic plasticity, neurogenesis, cortical reorganization, and functional network optimization.

Clinical implementation should prioritize early intervention initiation within the subacute post-stroke period to capitalize on heightened neuroplastic potential. Combined multimodal protocols leveraging synergistic mechanisms offer superior outcomes compared to monotherapies. Technology-enhanced interventions including virtual reality and computer-assisted training provide effective, scalable, and engaging rehabilitation options. Personalized medicine approaches integrating biomarker-guided treatment selection hold promise for optimizing individual outcomes.

The neuroplasticity paradigm fundamentally transforms our conceptualization of post-stroke cognitive rehabilitation from compensation for fixed deficits to active promotion of neural reorganization and functional restoration. As the field advances, integration of emerging neurotechnologies, precision medicine approaches, and mechanistic insights will enable increasingly effective, personalized interventions that harness the brain’s remarkable capacity for adaptation and recovery. Future research must address current knowledge gaps while translating promising findings into accessible, evidence-based clinical practice that improves outcomes for the millions of individuals affected by post-stroke cognitive impairment worldwide.

## Data Availability

The original contributions presented in this study are included in the article/Supplementary material, further inquiries can be directed to the corresponding author.

## Supplementary material

Supplementary Appendix 1 Complete database-specific search strategies. This supplementary appendix provides the complete, reproducible search strategies used for each of the four electronic databases (PubMed, Web of Science, Cochrane CENTRAL, and Embase). Search strategies include all search terms, Boolean operators, field tags, and filters applied. MeSH terms (PubMed), Emtree terms (Embase), and MeSH descriptors (Cochrane) were exploded to capture all relevant subheadings. All searches were conducted on 15 October 2025, with date restrictions from 1 January 2020 to 15 October 2025. A summary table of search yields by database is included.
