# Supplementary material for: Interventions for post-stroke cognitive impairment: a systematic review of neuroplastic mechanisms, comparative efficacy, and multimodal approaches
Source: Front Neurosci. 2026 Sep 9;20:1778597. doi: 10.3389/fnins.2026.1778597 (PMC13597674; doi:10.3389/fnins.2026.1778597)
Supplement: Supplementary file 1 [file Data_Sheet_1.docx]

**Supplementary Appendix S1**

**Complete Database-Specific Search Strategies**

**Manuscript:** *Interventions for Post-Stroke Cognitive Impairment: A Systematic Review of Neuroplastic Mechanisms, Comparative Efficacy, and Multimodal Approaches*

**Search date:** October 15, 2025

**Date range:** January 1, 2020 – October 15, 2025

**Language restriction:** English only

**Study design filter:** Randomized controlled trials

**1. PubMed (via NCBI) — Yield: 1,126 records**

*Search strategy using MeSH terms and free-text keywords. Filters applied: Randomized Controlled Trial; Publication date: 2020/01/01 – 2025/10/15; English.*

```
#1  "Stroke"[MeSH Terms]
#2  "Cerebrovascular Disorders"[MeSH Terms]
#3  "Brain Ischemia"[MeSH Terms]
#4  "Cerebral Hemorrhage"[MeSH Terms]
#5  stroke[Title/Abstract]
#6  "cerebrovascular accident"[Title/Abstract]
#7  "ischemic stroke"[Title/Abstract]
#8  "hemorrhagic stroke"[Title/Abstract]
#9  "cerebral infarction"[Title/Abstract]
#10  #1 OR #2 OR #3 OR #4 OR #5 OR #6 OR #7 OR #8 OR #9
#11  "Cognitive Dysfunction"[MeSH Terms]
#12  "Cognition Disorders"[MeSH Terms]
#13  "cognitive impairment"[Title/Abstract]
#14  "cognitive dysfunction"[Title/Abstract]
#15  "cognitive deficit*"[Title/Abstract]
#16  "post-stroke cognitive impairment"[Title/Abstract]
#17  PSCI[Title/Abstract]
#18  "vascular cognitive impairment"[Title/Abstract]
#19  "vascular dementia"[Title/Abstract]
#20  #11 OR #12 OR #13 OR #14 OR #15 OR #16 OR #17 OR #18 OR #19
#21  "Neuronal Plasticity"[MeSH Terms]
#22  "neuroplasticity"[Title/Abstract]
#23  "brain plasticity"[Title/Abstract]
#24  "neural plasticity"[Title/Abstract]
#25  "synaptic plasticity"[Title/Abstract]
#26  "cortical reorganization"[Title/Abstract]
#27  "cortical plasticity"[Title/Abstract]
#28  #21 OR #22 OR #23 OR #24 OR #25 OR #26 OR #27
#29  "Transcranial Direct Current Stimulation"[MeSH Terms]
#30  "tDCS"[Title/Abstract]
#31  "transcranial direct current stimulation"[Title/Abstract]
#32  "Transcranial Magnetic Stimulation"[MeSH Terms]
#33  "rTMS"[Title/Abstract]
#34  "repetitive transcranial magnetic stimulation"[Title/Abstract]
#35  "theta burst stimulation"[Title/Abstract]
#36  "brain stimulation"[Title/Abstract]
#37  "non-invasive brain stimulation"[Title/Abstract]
#38  "Cognitive Behavioral Therapy"[MeSH Terms]
#39  "cognitive training"[Title/Abstract]
#40  "cognitive rehabilitation"[Title/Abstract]
#41  "computer-assisted cognitive training"[Title/Abstract]
#42  "Virtual Reality"[MeSH Terms]
#43  "virtual reality"[Title/Abstract]
#44  "VR"[Title/Abstract] AND (cognitive[Title/Abstract] OR rehabilitation[Title/Abstract])
#45  "multimodal intervention"[Title/Abstract]
#46  "combined intervention"[Title/Abstract]
#47  "neuromodulation"[Title/Abstract]
#48  #29 OR #30 OR #31 OR #32 OR #33 OR #34 OR #35 OR #36 OR #37 OR #38 OR #39 OR #40 OR #41 OR #42 OR #43 OR #44 OR #45 OR #46 OR #47
#49  #10 AND #20 AND (#28 OR #48)
#50  #49 AND ("2020/01/01"[Date - Publication] : "2025/10/15"[Date - Publication])
#51  #50 AND "Randomized Controlled Trial"[Publication Type]
#52  #50 AND (randomized[Title/Abstract] OR randomised[Title/Abstract] OR "controlled trial"[Title/Abstract] OR RCT[Title/Abstract])
#53  #51 OR #52
#54  #53 AND English[Language]
```

*Final result (#54): 1,126 records*

**2. Web of Science (Core Collection) — Yield: 982 records**

*Search strategy using Topic (TS) field. Filters: Document Types = Article; Publication Years = 2020–2025; Languages = English.*

```
#1  TS=(stroke OR "cerebrovascular accident" OR "ischemic stroke" OR "hemorrhagic stroke" OR "cerebral infarction")
#2  TS=("cognitive impairment" OR "cognitive dysfunction" OR "cognitive deficit*" OR "post-stroke cognitive impairment" OR PSCI OR "vascular cognitive impairment")
#3  TS=(neuroplasticity OR "brain plasticity" OR "neural plasticity" OR "synaptic plasticity" OR "cortical reorganization")
#4  TS=("transcranial direct current stimulation" OR tDCS OR "repetitive transcranial magnetic stimulation" OR rTMS OR "theta burst stimulation" OR "brain stimulation" OR "non-invasive brain stimulation" OR "cognitive training" OR "cognitive rehabilitation" OR "virtual reality" OR "computer-assisted" OR "multimodal intervention" OR neuromodulation)
#5  #1 AND #2 AND (#3 OR #4)
#6  #5 AND TS=(randomized OR randomised OR "controlled trial" OR RCT OR "random allocation" OR "clinical trial")
#7  Refined by: Document Types = Article; Publication Years = 2020–2025; Languages = English
```

*Final result (#7): 982 records*

**3. Cochrane Central Register of Controlled Trials (CENTRAL) — Yield: 847 records**

*Search strategy using MeSH descriptors and free-text. Limited to Trials; Publication year: 2020–2025.*

```
#1  [mh "Stroke"] OR [mh "Cerebrovascular Disorders"] OR [mh "Brain Ischemia"] OR (stroke OR "cerebrovascular accident" OR "ischemic stroke" OR "hemorrhagic stroke" OR "cerebral infarction"):ti,ab,kw
#2  [mh "Cognitive Dysfunction"] OR [mh "Cognition Disorders"] OR ("cognitive impairment" OR "cognitive dysfunction" OR "cognitive deficit*" OR "post-stroke cognitive impairment" OR PSCI OR "vascular cognitive impairment"):ti,ab,kw
#3  [mh "Neuronal Plasticity"] OR (neuroplasticity OR "brain plasticity" OR "neural plasticity" OR "synaptic plasticity" OR "cortical reorganization"):ti,ab,kw
#4  [mh "Transcranial Direct Current Stimulation"] OR [mh "Transcranial Magnetic Stimulation"] OR [mh "Virtual Reality"] OR (tDCS OR rTMS OR "theta burst stimulation" OR "brain stimulation" OR "cognitive training" OR "cognitive rehabilitation" OR "virtual reality" OR "computer-assisted" OR "multimodal intervention" OR neuromodulation):ti,ab,kw
#5  #1 AND #2 AND (#3 OR #4)
#6  #5 with Cochrane Library publication date Between Jan 2020 and Oct 2025, in Trials
```

*Final result (#6): 847 records*

**4. Embase (via Elsevier) — Yield: 892 records**

*Search strategy using Emtree terms and free-text. Filters: Publication types = Article; Date range: 2020–2025; Language = English.*

```
#1  'cerebrovascular accident'/exp OR 'brain ischemia'/exp OR 'brain hemorrhage'/exp OR (stroke OR 'cerebrovascular accident' OR 'ischemic stroke' OR 'hemorrhagic stroke' OR 'cerebral infarction'):ti,ab
#2  'cognitive defect'/exp OR 'mild cognitive impairment'/exp OR ('cognitive impairment' OR 'cognitive dysfunction' OR 'cognitive deficit*' OR 'post-stroke cognitive impairment' OR PSCI OR 'vascular cognitive impairment'):ti,ab
#3  'neuroplasticity'/exp OR (neuroplasticity OR 'brain plasticity' OR 'neural plasticity' OR 'synaptic plasticity' OR 'cortical reorganization'):ti,ab
#4  'transcranial direct current stimulation'/exp OR 'transcranial magnetic stimulation'/exp OR 'virtual reality'/exp OR (tDCS OR rTMS OR 'theta burst stimulation' OR 'brain stimulation' OR 'non-invasive brain stimulation' OR 'cognitive training' OR 'cognitive rehabilitation' OR 'virtual reality' OR 'computer-assisted' OR 'multimodal intervention' OR neuromodulation):ti,ab
#5  #1 AND #2 AND (#3 OR #4)
#6  #5 AND ('randomized controlled trial'/exp OR 'randomization'/exp OR (randomized OR randomised OR 'controlled trial' OR RCT):ti,ab)
#7  #6 AND [2020-2025]/py AND [english]/lim
#8  #7 NOT ('conference abstract'/it OR 'conference paper'/it OR 'editorial'/it OR 'letter'/it OR 'note'/it)
```

*Final result (#8): 892 records*

**Summary of Search Results**

| **Database** | **Records Retrieved** |
| --- | --- |
| PubMed | 1,126 |
| Web of Science | 982 |
| Cochrane CENTRAL | 847 |
| Embase | 892 |
| **Total** | **3,847** |

**Additional Search Methods**

In addition to the electronic database searches described above, the reference lists of all included studies and relevant systematic reviews identified during the search process were manually screened to identify any additional eligible publications not captured by the electronic search. No additional studies were identified through this supplementary search method.

**Search Strategy Notes**

1. All searches were conducted on October 15, 2025.

2. Search strategies were developed by the author (Y.Z.) with reference to published search filters for randomized controlled trials and systematic review methodology guidelines.

3. MeSH terms (PubMed), Emtree terms (Embase), and MeSH descriptors (Cochrane) were exploded to capture all relevant subheadings.

4. Truncation (*) was used where appropriate to capture variant word endings.

5. The search was restricted to English-language publications due to resource constraints for translation.

6. No restrictions were applied based on country of origin, sample size, or publication status (beyond peer-reviewed journals).

7. Grey literature, conference abstracts, dissertations, and preprints were excluded.
